# Carbon nanotubes affect the toxicity of CuO nanoparticles to denitrification in marine sediments by altering cellular internalization of nanoparticle

**DOI:** 10.1038/srep27748

**Published:** 2016-06-09

**Authors:** Xiong Zheng, Yinglong Su, Yinguang Chen, Rui Wan, Mu Li, Haining Huang, Xu Li

**Affiliations:** 1 State Key Laboratory of Pollution Control and Resource Reuse, School of Environmental Science and Engineering, Tongji University, 1239 Siping Road, Shanghai 200092, China; 2 Department of Civil Engineering, University of Nebraska-Lincoln, 844 North 16th Street, Lincoln, Nebraska 68588-6105, United States

## Abstract

Denitrification is an important pathway for nitrate transformation in marine sediments, and this process has been observed to be negatively affected by engineered nanomaterials. However, previous studies only focused on the potential effect of a certain type of nanomaterial on microbial denitrification. Here we show that the toxicity of CuO nanoparticles (NPs) to denitrification in marine sediments is highly affected by the presence of carbon nanotubes (CNTs). It was found that the removal efficiency of total NO_X_^−^-N (NO_3_^−^-N and NO_2_^−^-N) in the presence of CuO NPs was only 62.3%, but it increased to 81.1% when CNTs appeared in this circumstance. Our data revealed that CuO NPs were more easily attached to CNTs rather than cell surface because of the lower energy barrier (3.5 versus 36.2 kT). Further studies confirmed that the presence of CNTs caused the formation of large, incompact, non-uniform dispersed, and more negatively charged CuO-CNTs heteroaggregates, and thus reduced the nanoparticle internalization by cells, leading to less toxicity to metabolism of carbon source, generation of reduction equivalent, and activities of nitrate reductase and nitrite reductase. These results indicate that assessing nanomaterial-induced risks in real circumstances needs to consider the “mixed” effects of nanomaterials.

Microbial denitrification has been regarded as an important process involved in global climate, water quality, and soil fertility^1^. According to this pathway, nitrate can be sequentially reduced to nitrite, nitric oxide, nitrous oxide, and dinitrogen gas^2^. In recent years, man-made nanomaterials are widely used in various fields, such as biomedicine, material synthesis, and chemical catalysis, due to their outstanding properties^3^,^4^. However, the increasing manufacture and utilization of these nanomaterials inevitably lead to their releases into the environment, which poses the potential risks to the denitrification process^5^,^6^,^7^. For example, the presence of quantum dots was found to negatively affect the activity of denitrifying microorganism^8^, whereas ZnO nanoparticles (NPs) caused significant inhibition to denitrification of both pure culture and mixed culture^9^,^10^. Clearly, previous studies focused on the possible influence of single type of nanomaterial on microbial denitrification. It should be noted that different types of nanomaterials usually coexist in real circumstances^11^. Therefore, when we assess the nanomaterial-induced risks in real circumstances, the “mixed” effects of nanomaterials need to be considered.

The cytotoxicity of nanomaterials can be affected by their properties^12^ and the interactions between cells and nanomaterials^13^. The literature has reported that engineered nanomaterials, such as CNTs, ZnO, and silver NPs, show severe impacts on cell membrane integrity and microbial activity, because they might easily enter cells or release toxic metal ions^14^,^15^,^16^,^17^,^18^. Meanwhile, the geometry morphology (such as compactness) of nanomaterial is also related to its cytotoxicity^19^. Previous studies observed that the features of nanomaterials, such as particle size and geometry, are easily changed with the variations of environmental conditions^20^,^21^. Therefore, it can be anticipated that the presence of one type of nanomaterial might affect the characteristics of another when both NPs are present in the same circumstance. However, to date, it is largely unknown whether the coexistence of different nanomaterials could affect the toxicity of single nanomaterial to microbial denitrification in marine sediments.

Carbon nanotubes, a widely used class of carbon-based nanomaterials, have been shown to be able to cause negative effects on bacterial viability^22^, soil microbial communities^7^, and wastewater treatment process^23^, and their toxicities were determined by functionalization, size, and morphology^14^,^21^. Also, CuO NPs were reported to inhibit the denitrification efficiency of microbe^24^, mediate DNA damage^25^, or cause genotoxicity^26^. Nevertheless, once CNTs and CuO NPs co-existed in the environment, their potential environmental risks remain unclear. Here we report the potential effects of CuO NPs, CNTs, and CuO NPs + CNTs on denitrification in marine sediments. Then, the mechanisms for the different toxicities of single (CuO NPs or CNTs) and mixed (CuO NPs + CNTs) nanomaterials to microbial denitrification were explored by analyzing interacting energy, nanomaterial property (including particle polydispersity index, fractal dimension, and electrical property), cell cytoplasm density, carbon source metabolism, reduction equivalent generation, and key enzymes activities. The data from this work show the importance of the “mixed” effects of nanomaterials in real circumstances, when we assess the nanomaterial-induced environmental risks.

## Results

### CNTs affect the toxicity of CuO NPs to denitrification in marine sediments

**Figure 1:**
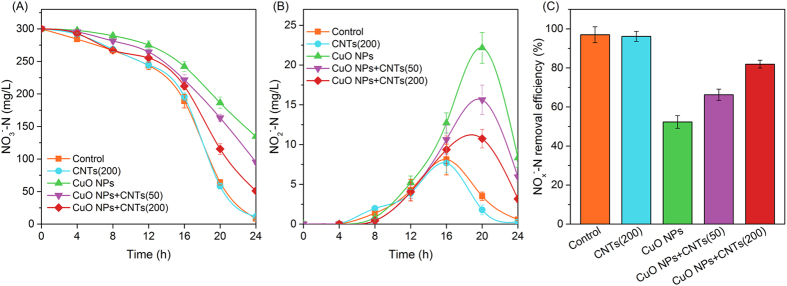
CNTs affect the toxicity of CuO NPs to denitrification in marine sediments. (**A–C**) Show the variations of NO_3_^−^-N, the accumulation of NO_2_^−^-N, and the final total NO_X_^−^-N removal efficiency, respectively. Error bars represent standard deviations of triplicate tests.

Figure 1 illustrates the denitrification performances with single CuO NPs, single CNTs and CuO NPs + CNTs present in marine sediments, respectively. It can be found that the variations of nitrate in the single CNTs tests were almost the same as those in the control. Nevertheless, the concentration of nitrate was significantly higher in the CuO NPs tests than in the control (*p* < 0.05), which indicated that the reduction of nitrate was significantly inhibited by CuO NPs. When 50 mg/g of CNTs were present in the CuO NPs system, the final nitrate concentration, compared with single CuO NPs, was remarkably decreased from 102.6 to 72.9 mg/L, which was further declined to 52.4 mg/L with the increase of CNTs to 200 mg/g. Meanwhile, the data in Fig. 1B implied that more nitrite accumulation was caused by single CuO NPs, and the final nitrite concentration was 10.66 mg/L, which was 8 times of the control or 9 times of single CNTs. However, the presence of CNTs significantly alleviated the impacts of CuO NPs on nitrite accumulation, and the final nitrite was respectively 6.67 and 4.27 mg/L with the supplement of 50 and 200 mg/g of CNTs. Figure 1C shows that the final removal efficiencies of total NO_X_^−^-N (NO_3_^−^-N and NO_2_^−^-N) in single CuO NPs, single CNTs and control tests were 62.3%, 91.1% and 95.0%, respectively, which became to 73.5% or 81.1% after 50 or 200 mg/g CNTs were present in the CuO NPs system. Clearly, the presence of CNTs reduced the toxicity of CuO NPs to denitrification in marine sediments.

### CNTs decrease the CuO NPs-induced effects on carbon source utilization, reduction equivalent generation and denitrifying enzyme activity

**Figure 2:**
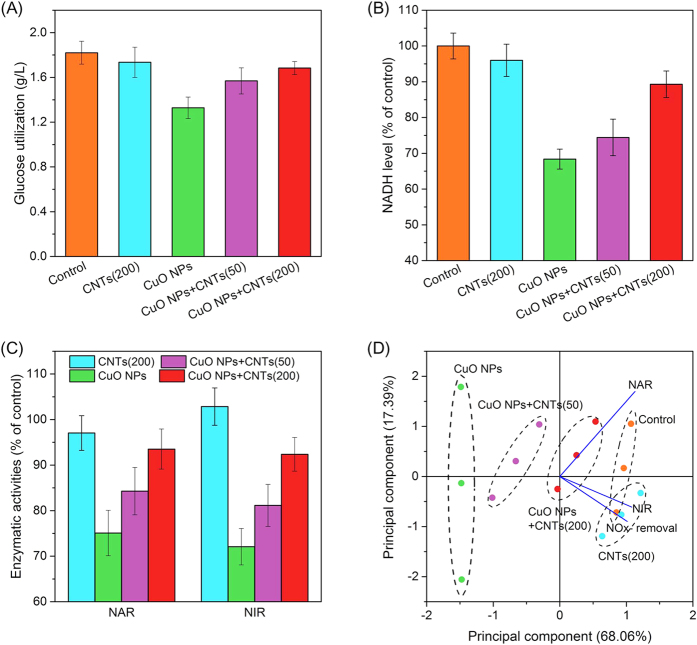
Effects of CNTs on the toxicity of CuO NPs to the glucose consumption (**A**), the NADH generation (**B**), the denitrifying enzyme activities (**C**), and the principal component analysis of NOx^−^-N removal efficiency, NAR and NIR under different experimental conditions (**D**). Error bars represent standard deviations of triplicate tests.

Denitrifying bacteria need to utilize carbon source (such as glucose) to provide energy for cell growth and produce reduction equivalent (i.e., NADH) for nitrate and nitrite bio-reductions. As shown in Fig. 2A, bacteria with CuO NPs utilized 1.33 g/L of glucose, which was significantly lower than that in the control (1.82 g/L) and that with CNTs (1.74 g/L). However, when 50 and 200 mg/g of CNTs were present in the CuO NPs system, the utilized glucose was increased to 1.57 g/L and 1.68 g/L, respectively. During the metabolism of glucose, NADH is produced, and then served as the reduction equivalent for denitrification^27^. The data in Fig. 2B revealed that CuO NPs decreased the intracellular NADH level to 68% of the control. However, the NADH content was respectively recovered to 74% and 89% of the control after the addition of 50 and 200 mg/g of CNTs.

The bio-reduction of nitrate to dinitrogen is catalyzed by a series of reductases, and the denitrification performance can be affected once the enzymatic activities are inhibited^9^. As seen in Fig. 2C, single CuO NPs caused significantly inhibitory effect on the activity of nitrate reductase (NAR) (75.1% of the control), and the presence of 50 and 200 mg/g of CNTs led to the recovery of NAR activity to 84.3% and 93.5% of the control, respectively. Likewise, the activity of nitrite reductase (NIR) was increased from 72.1% to 81.2% and 92.4% of the control with increasing the CNTs concentrations from 0 to 50 and 200 mg/g, indicating that CNTs also reduced the negative influence of CuO NPs on the activity of NIR. Figure 2D illustrates the principal component analysis (PCA) of the correlation of NO_x_^−^-N removal efficiency and activities of NAR and NIR under different exposure conditions, and the data indicated that the activity of NIR showed better association with NO_x_^−^-N removal than that of NAR.

### CNTs reduce the cellular internalization of CuO NPs during denitrification

**Figure 3:**
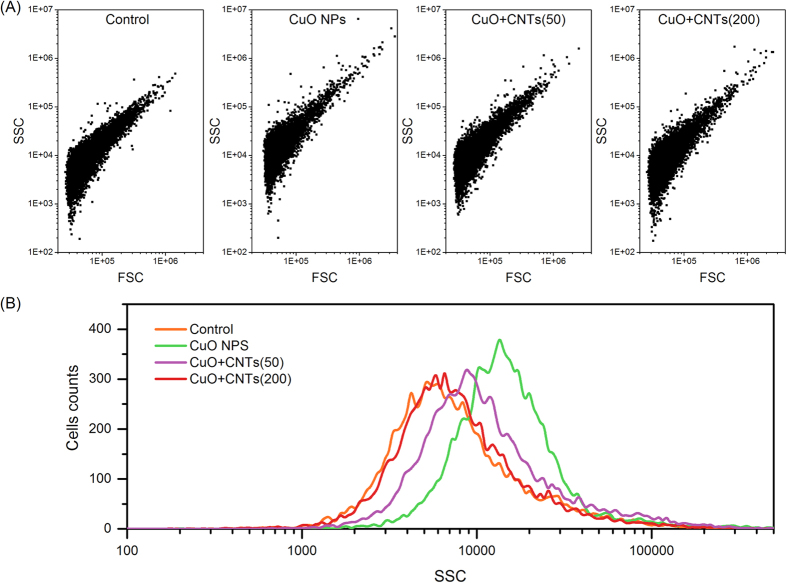
Scatter light analysis of nanoparticles uptake by bacteria via a flow cytometry. (**A**) FSC and SSC intensity distributions of cells under different exposure conditions; (**B**) Side scatter statistics analysis of cells exposed to CuO NPs, CNTs, and mixed CuO + CNTs.

The uptake of nanoparticle by cells is closely related to the NPs-induced toxicity. Thus, the cellular internalization of CuO NPs was investigated by flow cytometry in this study^20^, and the analysis of side scatter (SSC) in flow cytometry was used to measure the uptake of nanoparticle by bacterial cells^28^. Figure 3 shows the dot plots of forward scatter (FSC) and SSC of cells under different conditions. Compared with the control, there was no significant change in the SSC of the single CNTs tests (data not shown). However, the SSC of cells exposed to CuO NPs was increased to 1.62 folds of the control according to the SSC distribution (Fig. 3B). More importantly, the presence of 50 and 200 mg/g of CNTs in the CuO NPs system decreased the SSC to 1.33- fold and 1.16- fold of the control, respectively. Therefore, although CuO NPs were taken up by cells, the presence of CNTs could reduce the cellular internalization of nanoparticle.

### Influence of CNTs on the interaction between CuO NPs and cells

**Figure 4:**
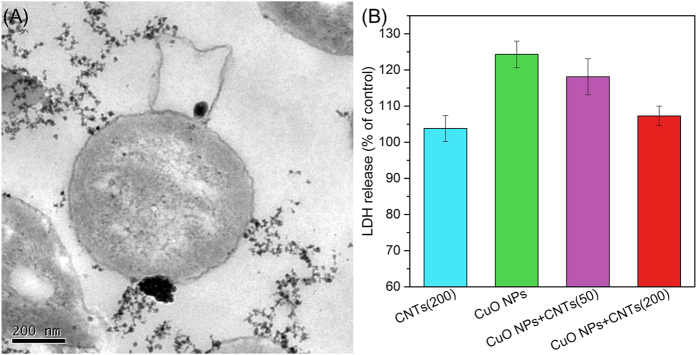
TEM images (**A**) and LDH release (**B**) of denitrifying bacteria after exposure to CuO NPs, CNTs, and CuO NPs + CNTs. Error bars represent standard deviations of triplicate tests.

Before CuO NPs were taken up by cells, they first needed to contact with the surface of microbe. As shown in Fig. 4A, CuO NPs tended to attach onto CNTs other than cells, suggesting that the presence of CNTs was likely to block the entrance of CuO NPs into cells. Previous studies reported that the entrance of nanoparticle would cause cell membrane damage, which subsequently resulted in the leakage of cytoplasm substances, such as the rise of extracellular LDH level^26^. Figure 4B demonstrates that CNTs did not cause significant LDH release, which was consistent with our previous observation^29^. However, CuO NPs led to the damage of membrane integrity for significantly elevating the released LDH (124% of control). When 50 and 200 mg/g of CNTs were present in the CuO NPs system, the levels of LDH release were decreased to 118% and 107% of the control, respectively, indicating that the presence of CNTs alleviated the membrane damage induced by CuO NPs.

## Discussion

The literature reported that the cytotoxicity of CuO NPs to algae^30^, mussel^31^, or human cells^26^ was attributed to nanoparticle itself, because CuO NPs showed distinct modes of action and greater magnitude influence compared with dissolved Cu^2+^. However, in other studies on murine microalgae^32^, macrophage cells^33^, and human lung cells^34^, the toxic effects of CuO NPs were resulted from the dissolved Cu^2+^. In this study, we also considered the dissolution feature of CuO NPs and the toxicity of the corresponding dissolved ions. Figure S1A (Supplementary Information) shows that the final released Cu^2+^ was 0.565 mg/L in the absence of CNTs, which only accounted for less than 6% of 10 mg/L of CuO NPs. The similar observations were made by the previous studies^25^,^30^. Nevertheless, the observed concentrations of Cu^2+^ in the presence of 50 and 200 mg/L of CNTs were 0.4967 and 0.3629 mg/L, respectively. Figure S1B (Supplementary Information) illustrates the influences of the released Cu^2+^ on the sedimentary denitrification. It can be seen that the removal efficiencies of NO_x_^−^-N in the absence and presence of 0.3629 mg/L of Cu^2+^ were 95.0% and 96.4%, respectively. With the increase of Cu^2+^ to 0.4967 and 0.5654 mg/L, the NO_x_^−^-N removal efficiency was slightly changed to 93.3% and 90.1%, respectively. The statistical analysis indicated that the influence of the released Cu^2+^ in the range of 0 to 0.5654 mg/L on the NO_x_^−^-N removal was insignificant (*p* > 0.05). These results confirmed that it was not the released ions but the nanoparticles themselves accounting for the toxicity of CuO NPs to denitrification in marine sediments.

To investigate the underlying mechanism, a widely used model denitrifier, *Paracoccus denitrificans*, was used in this study. It was observed that the presence of CNTs also alleviated the negative influence of CuO NPs on the denitrification performance of *P. denitrificans* (Figure S2, Supplementary Information), which was similar to that of sedimentary denitrification. As seen in Figure S3 (Supplementary Information), CuO NPs induced 35.7% of the loss of cell viability, whereas the presence of 50 and 200 mg/g of CNTs decreased the loss of viability (28.2% and 7.2%). The data of Fig. 2 further revealed that the reason for CNTs alleviating the toxicity of CuO NPs to denitrification was attributed to the improved glucose utilization, which caused cell growth increase and more reduction equivalent generation. Meanwhile, the presence of CNTs significantly attenuated the effects of CuO NPs on denitrifying enzymes, and the influence on the activity of NIR showed the higher correlation with the performance of NO_x_^−^-N removal.

**Figure 5:**
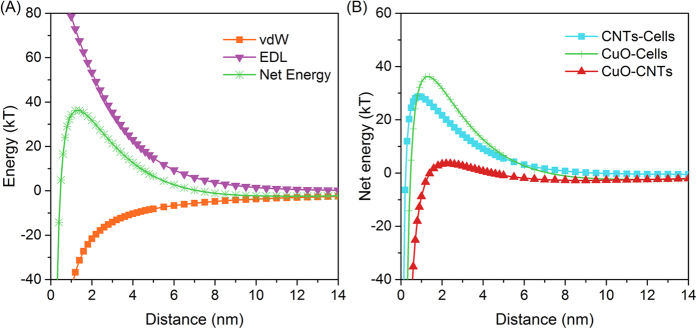
Interfacial interaction between CuO NPs and cells expressed by van der Waals (vdW), electrostatic double layer (EDL) and net energy (**A**), and net energy comparison among CNTs-Cells, CuO-Cells, and CuO-CNTs (**B**).

Our data confirmed that the presence of CNTs reduced the cellular internalization of CuO NPs (Fig. 3). To explore the reason of CNTs decreasing the contact between CuO NPs and cells, the interacting energy (including van der Waals attraction, electrostatic double layer energy, and the total energy) between CuO NPs and cells was calculated and shown in Fig. 5. Because the measured zeta potentials of CuO NPs and cells were negative (−14.7 mV versus −21.5 mV), the electrostatic double layer force showed the repulsion interaction, which prevented the contact between CuO NPs and cells (Fig. 5A). The van der Waals force exhibits attractive interaction. The net energy is the sum of van der Waals and electrostatic double layer, and the maximum of net energy has been regarded as the energy barrier to overcome prior to getting close, which determined the contacting possibility of particles^13^. Figure 5B depicts that the energy barrier was 36.2, 28.7, and 3.5 kT for the pairs of CuO-Cells, CNTs-Cells, and CuO-CNTs, respectively. These results suggested that CuO NPs were most likely to contact with CNTs rather than cells, and the contacting probability of pairs was in the following order: CuO-CNTs > CNTs-Cells > CuO-Cells.

**Figure 6:**
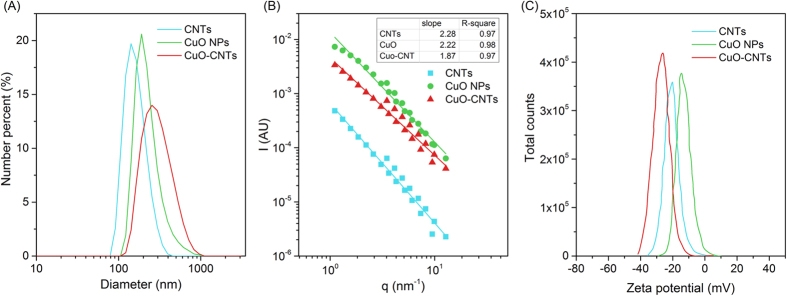
Size distribution (**A**), fractal dimension (**B**), and zeta potential distribution (**C**) of CNTs, CuO NPs, and CuO-CNTs.

Since CNTs tended to interact with CuO NPs, it might alter the surface properties. The changes in nanomaterial properties have been reported to influence its cellular uptake and subsequent biological effect^35^. In this study, the average hydrodynamic diameters of CuO NPs and CNTs were 228 and 193 nm, respectively, but it became 268 nm in the co-existence system of CuO NPs + CNTs, indicating the formation of large particles and aggregates. Furthermore, the disperse property of nanoparticles evaluated by polydispersity index (PDI) showed that the mixed nanoparticles exhibited greater PDI (0.478) than their individuals (0.236 with CuO NPs and 0.197 with CNTs) in the reaction medium (Fig. 6A). Broader size distribution with higher PDI value indicated more polydispersed samples^21^. Therefore, the presence of CNTs in the CuO NPs system caused a more polydispersed status of nanoparticles.

The structural morphology of nanoparticles was investigated by static light scattering technique, which was used to track colloidal aggregation and aggregation state^36^. As seen in Fig. 6B, the calculated *D*_*f*_ values of CNTs, CuO NPs, and CuO-CNTs were 2.28, 2.22, and 1.87, respectively. Generally, the *D*_*f*_ values of colloidal aggregates fall in the range of 1 to 3, and higher values are associated with more compact morphological structures^37^. Apparently, the formed aggregation of CuO-CNTs possessed looser structure than that of CuO NPs. Figure 6C demonstrates that the heteroaggregates of CuO-CNTs had more negative surface charge for the zeta potential (−27.5 mV) being lower than that of CuO NPs (−14.7 mV) or CNTs (−21.1 mV). These data revealed that the presence of CNTs led to the formation of large, non-uniformed and incompact heteroaggregates with greater negative surface charge. Because the greater size or more negatively charged nanoparticles were less taken up by cells^38^,^39^,^40^, it can be inferred that the CuO-CNTs heteroaggregates were difficult to be internalized by bacterial cells, which finally reduced the toxicity of CuO NPs to denitrification.

In summary, the data of this work indicate that the presence of CNTs highly affects the toxicity of CuO NPs to denitrification in marine sediments via altering the cellular internalization of nanoparticle and relieving the negative influences on the intracellular metabolism and the key enzyme activity of denitrifers, such as glucose utilization, reduction equivalent generation, and denitrifying enzymes. In the future, more attention should be paid to determine the potential effects of mixed nanomaterials on the way of nanoparticle entering cells and the interaction between nanoparticles and intracellular biomacromolecules. These investigations will help understand the biological responses of microbiota to “mixed” nanomaterials in real circumstances.

## Methods

### Preparation of nanomaterials

CuO NPs (primary particle size <50 nm) and CNTs were purchased from Sigma-Aldrich (St. Louis, MO, USA) and Nanjing XF Nanotech Port Co. Ltd. (China), respectively. Before experiments, CNTs were treated according to the literature^21^. Firstly, CNTs were heated at 350 °C for 3 h. After being cooled down, nanotubes were placed in 12 M HCl for 8 h, bath sonicated under ambient conditions for 1 h, and then washed by copious amounts of Milli-Q water until neutral pH. Finally the powder CNTs were gotten after being dried in an oven (60 °C) overnight. The stock nanomaterial suspension was prepared by ultrasonicating the powder CuO NPs or CNTs in Milli-Q water for 1 h.

### Source of denitrifiers

Marine sediments were used as the microbiota source of sedimentary denitrification, which was collected from the Chongming Island (31° 30′ 26″ N, 121° 58′ 51″ E near the East Sea), Shanghai, China, and then sieved through 100-mesh sieve. After the determination of suspended solid concentration, the sediment was rinsed with 10 mM phosphate buffer (pH 7.4) for 3 times for the following exposure experiments. Also, *Paracoccus denitrificans* (ATCC 19367), purchased from American Type Culture Collection, was chosen as a model denitrifying bacteria to investigate the mechanisms due to its wide existence in sediment^41^. Prior to experiments *P. denitrificans* was grown in Difco nutrient broth at 30 °C overnight, harvested in midexponential phase, and then used as the inoculum for the following experiments.

### Investigation of CNTs affecting the toxicity of CuO NPs to denitrification

To sedimentary denitrification tests, sedimentary microbiota was respectively exposed to single CuO NPs (10 mg/g·(suspended solid (SS)), single CNTs (200 mg/g·SS), mixed CuO NPs + CNTs (10 + 50 mg/g·SS), and mixed CuO NPs + CNTs (10 + 200 mg/g·SS) in a modified artificial seawater medium, which contained (g/L): glucose, 5.0; NaCl, 19.6; KCl, 0.63; CaCl_2_·2H_2_O, 1.4; MgSO_4_·7H_2_O, 6.29; MgCl_2_·6H_2_O, 4.66; KNO_3_, 2.16, and trace elements solution of 1.0 mL/L^42^. The trace elements contained (g/L): FeSO_4_·7H_2_O, 2.50; MnCl_2_·4H_2_O, 0.02; ZnCl_2_, 0.34; Na_2_MoO_4_·2H_2_O, 0.242; CuCl_2_·2H_2_O, 0.135; and EDTA-Na_2_, 7.30. To model bacteria denitrification tests, *P. denitrificans* was respectively exposed to single CuO NPs (10 mg/g·dry cell weight (DCW)), single CNTs (200 mg/g·DCW), mixed CuO + CNTs (10 + 50 mg/g·DCW), and mixed CuO + CNTs (10 + 200 mg/g·DCW) in mineral medium, which had the following constitutions (g/L): glucose, 5.0 ; K_2_HPO_4_, 7.0; KH_2_PO_4_, 3.0; sodium citrate·2H_2_O, 0.5; MgSO_4_·7H_2_O, 0.1; (NH_4_)_2_SO_4_, 1.0; KNO_3_, 2.16; and trace elements solution of 50 μL/L^43^. Both medium were prepared in serum bottles, and placed into an autoclave for high-pressure sterilization (121 °C and 20 min). Then, in an anaerobic chamber, the stock suspensions of nanomaterials were added to the medium, followed by inoculating sedimentary inocula (1 g/L) or *P. denitrificans* (OD_600_ of 0.05). All serum bottles were sealed and placed in a shaker (160 rpm) with constant temperature of 30 °C, and the concentrations of NO_3_^−^-N, NO_2_^−^-N, and glucose were measured at intervals of 4 h for a total 24 h time according to our previous publication^9^.

### Calculation of interfacial interactions between nanomaterials and cells

The interaction between particles and model denitrifying bacteria (*P. denitrificans*) was evaluated by the DLVO theory, which described the total interaction as the combination of van der Waals (vdW) and electrostatic double layer (EDL) interaction^44^. The vdW interaction is attractive while EDL interaction can be repulsive in some cases, and both vary with the distance of particles surface (h). The total interaction energy (*V*_*T*_) is the sum of these two interactions, which determines whether the net interaction between particles is repulsive or attractive^45^,^46^. According to DLVO theory, the following relationship can be derived:


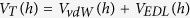



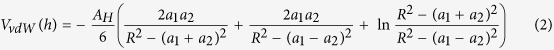


where *A*_*H*_ is the particle-particle Hamaker constant, *R* is the center distance between particles, *h* is the surface distance, and *a*_1_ and *a*_2_ are the radii. As to *A*_*H*_, values of 6.95 × 10^−21^, 1.57 × 10^−20^, and 3.11 × 10^−21^ were used for CuO-Cells, CuO-CNTs, and CNTs-Cells respectively, which were calculated according to literature^13^.

The EDL energy is calculated as following:


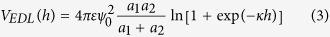


where *ε* is the permittivity of water, *ψ*_*0*_ is surface potential, and *κ* is the inverse Debye length which is expressed as:


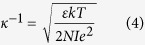


where *k* is the Boltzmann constant, *T* is the absolute temperature (K), *N* is the Avogadro’s number, *I* is ionic strength, and *e* is the unit charge.

### Analysis of nanomaterials characteristics

Dynamic light scattering (DLS) was used to measure the hydrodynamic diameter and zeta potential, and the data were collected on Zetasizer NanoZS (Malvern, UK). CuO NPs, CNTs and mixed CuO-CNTs suspensions were prepared in mineral media by ultrasonication for 1 h, and then the samples were used for measurement. The average diameter, polydispersity index (PDI) and zeta potential distribution were analyzed by Zetasizer Software v7.02 (Malvern, UK).

To determine the structural morphology of nanomaterials, static light scattering (SLS) was employed to obtain the fractal dimension (*D*_*f*_). The samples were prepared as above and detected on a Mastersizer 3000 (Malvern, UK). The scattered light intensity (*I*) is a function of *q* and fractal dimension *D*_*f*_ :


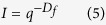


where *q* is the wave vector defined as:


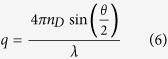


with *n*_*D*_ being the solvent index of refraction, *λ* the incident light wavelength, and *θ* the scattering angle. *I(q)* was linear on a log-log scale, and the *D*_*f*_ was extracted from the slope of the line which fitted the *I-q* data.

### Evaluation of cellular uptake of nanomaterials

Side scatter (SSC) light was carried out using a BD Accuri C6 (BD Bioscience, USA) as described in literatures^20^,^28^,^47^. The nanoparticles located inside cells would increase the refractive index, which implied the increment of side scatter light intensity^48^. *P. denitrificans* cells were treated with single CuO NPs (10 mg/g·DCW) and mixed nanomaterials (CuO NPs (10 mg/g·DCW) + CNTs (50 or 200 mg/g·DCW)) for 24 h in the medium, and then the cells were trypsinized, centrifuged at 3000 rpm for 10 min, and resuspended in 1 mL of medium for flow cytometry detection. A BD Accuri C6 (BD Biosciences, USA) contained 488 nm laser, forward scatter (FSC) diode detector and SSC detector. During the detection process, 10000 cells were collected and the FSC and SSC intensities were acquired. As each cell intercepts the path of laser beam, FSC intensity indicates cell size, and SSC is related to intracellular density. After being treated with nanoparticles, the increment in cellular SSC reflects the uptake potential of nanoparticles by cells.

### Measurements of nitrate reductase and nitrite reductase

At the end of the experiments, bacterial samples were harvested by centrifugation (5000 rpm for 10 min), and then washed with 100 mM potassium phosphate buffer (pH 7.4), and resuspended in the same buffer. The suspension was ultrasonicated (4 °C, 20 kHz) for 5 min, followed by centrifugation (16000 rpm) for 10 min to remove debris, and then the supernatant was used for the determination of enzymatic activity and protein immediately. The 2 mL of assay mixture contained 10 mM potassium phosphate buffer (pH 7.4), 1 mM methyl viologen, 5 mM Na_2_S_2_O_4_, and 1 mM reaction substrate (KNO_3_ or NaNO_2_), and the reaction was initialized by addition of 0.3 mL cell extracts. After 30 min of reaction in a 30 °C incubator, the concentrations of NO_3_^−^-N and NO_2_^−^-N were measured^9^, and the specific enzyme activity was calculated as μmol substrate/(min·mg protein).

### Measurement of reduction equivalent

The reduction equivalent was evaluated by intracellular nicotinamide adenine dinucleotide (NADH) level, and the detailed procedure of measurement was according to the literature^49^. At the end of the experiments samples samples were centrifuged at 15000 rpm for 1 min. The supernatant was removed, and 300 μL of 0.2 M NaOH was added to re-suspend the pellets. Then the samples were placed in a 50 °C water bath for 10 min, following on ice to cool down to 0 °C. The extracts were neutralized by adding 300 μL of 0.1 M HCl dropwise while vortexing. After centrifugation at 15000 rpm for 5 min, supernatants were transferred to new tubes for NADH concentration measurement by a cycling assay^50^. The reagent mixture contained equal volumes of 1.0 M Bicine buffer (pH 8.0), ethanol, 40 mM EDTA (pH 8.0), 4.2 mM thiazolyl blue (MTT), and twice the volume of 16.6 mM phenazine ethosulfate (PES), and then incubated at 30 °C for 10 min. The reaction mixture was prepared by following volumes: 50 μL neutralized cell extract, 0.3 mL distilled water, and 0.6 mL reagent mixture. The reaction was started by adding 50 μL of alcohol dehydrogenase (ADH, 500 U/mL), and then the absorbance at 570 nm was recorded for 10 min at 30 °C. The concentration of NADH was calibrated with standard solutions of NADH, and the final NADH level was calculated as per milligram of protein.

### Transmission electron microscopy analysis

Transmission electron microscopy was used to show the intracellular presence of nanoparticles by a Tecnai G2 spirit Biotwin (FEI, USA) with 120 kV accelerating voltage, and the samples were prepared as the literature^30^. The bacterial cells treated with or without nanomaterials for 24 h, and then fixed using 2.5% glutaraldehyde. The samples were further postfixed in 1% osmic acid for 1 h, washed with 0.1 M phosphate buffer solution (pH 7.4) for 3 times, and dehydrated in increasing concentration of acetone (30%, 50%, 70%, 90% and 100%) for 20 min each time. Then, samples were permeated and impregnated for 5 h at 60 °C, and the 60 nm ultrathin sections were made on Ni grids for subsequent imaging.

### Lactate dehydrogenase release assay

Membrane integrity was determined by lactate dehydrogenase (LDH) release using cytotoxicity detection kit (Roche Applied Science). After exposed to nanomaterials, the culture supernatants were seeded in a 96-well plate, then 50 μL of reaction mixture was added for 30 min incubation at 30 °C. The data were obtained at 490 nm absorbance on a microplate reader (BioTek, USA), and the results were expressed as a percentage of the control. A significant increase in LDH level would indicate cellular disruption.

### Cell viability assay

Cell viability was measured by Cell Counting Kit-8 (Dojindo) according to the manufacturer’s instructions. Briefly, after 24 h exposure, the cells were collected and placed in the 96-well plate, and then incubated with 10 μL of CCK-8 for 30 min. The absorbance was recorded at 450 nm in a microplate reader (BioTek, USA), and the results were calculated as the relative activity based on sample without nanoparticles treatment.

### Statistical analysis

In this study, all tests were performed in triplicate, and the results were expressed as mean ± standard deviation. The test of significance was carried out by analysis of variance and *p* < 0.05 was considered to be significantly different. Principal component analysis (PCA) was performed using XLSTAT.

## Additional Information

**How to cite this article**: Zheng, X. *et al.* Carbon nanotubes affect the toxicity of CuO nanoparticles to denitrification in marine sediments by altering cellular internalization of nanoparticle. *Sci. Rep.* **6**, 27748; doi: 10.1038/srep27748 (2016).

## Supplementary Material

Supplementary Information
