# Supplementary material for: Carbon nanotubes affect the toxicity of CuO nanoparticles to denitrification in marine sediments by altering cellular internalization of nanoparticle
Source: Sci Rep. 2016 Jun 9;6:27748. doi: 10.1038/srep27748 (PMC4899749; doi:10.1038/srep27748)
Supplement: Supplementary Information [file srep27748-s1.doc]

**Supplementary Information**

**Carbon nanotubes affect the toxicity of CuO nanoparticles to denitrification in marine sediments by altering cellular internalization of nanoparticle**

Xiong Zheng^1^, Yinglong Su^1^, Yinguang Chen^1^*, Rui Wan^1^, Mu Li^1^, Haining Huang^1^, Xu Li^2^

**Affiliation:**

^1^ State key laboratory of pollution control and Resource reuse, School of Environmental Science and Engineering, Tongji University, 1239 Siping Road, Shanghai 200092, China

^2^ Department of Civil Engineering, University of Nebraska-Lincoln, 844 North 16th Street, Lincoln, Nebraska 68588-6105, United States

**Corresponding author:**

*Correspondence to: Y. Chen (yg2chen@yahoo.com)

**Contents:**

Supplementary Figures S1-S3 2-4

Supplementary Methods 5

**Supplementary Figure S1**

**
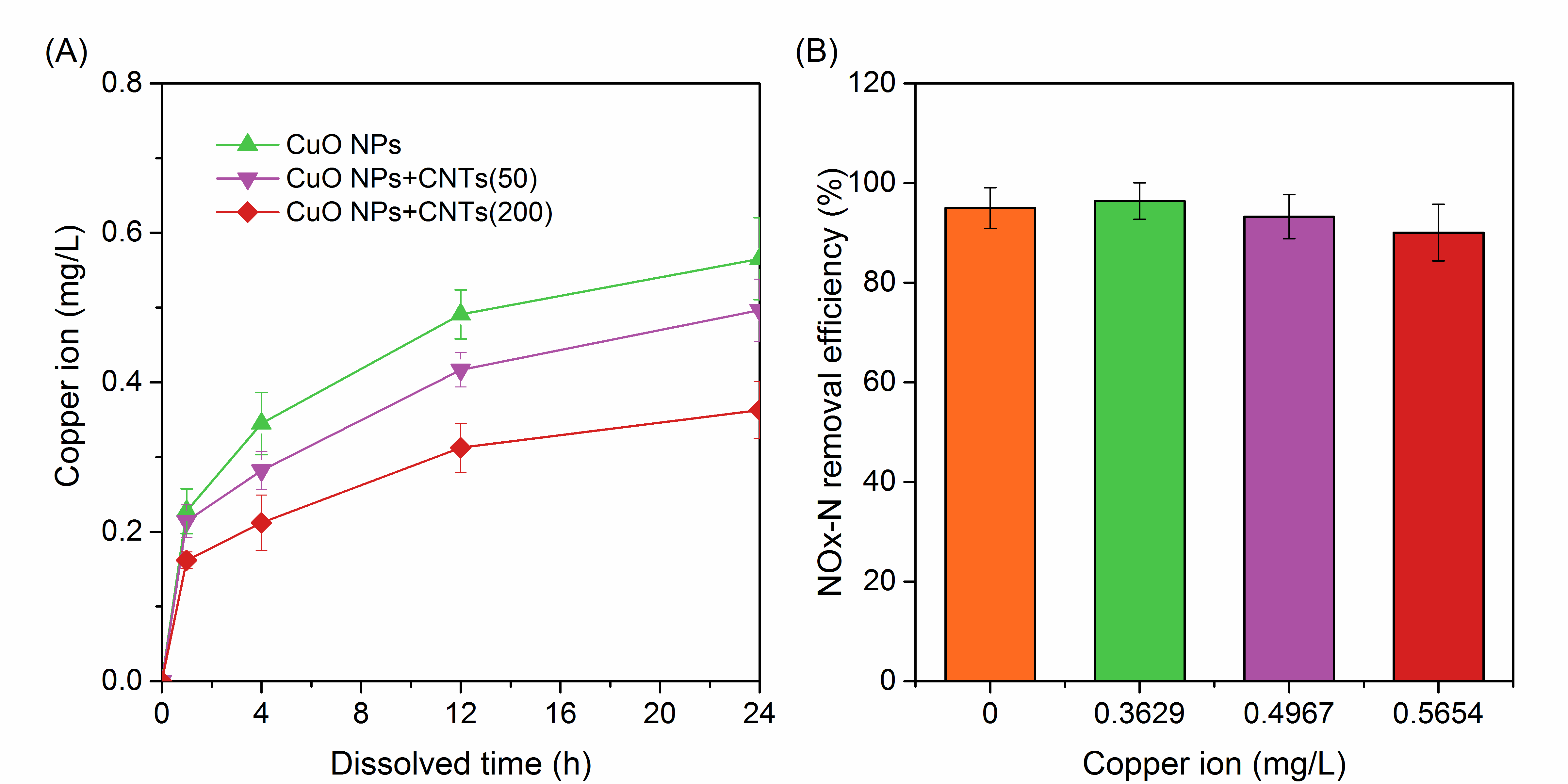
**

**Figure S1 | Dissolution pattern and toxicity of Cu^2+^ from CuO NPs.** **A**, The Cu^2+^ released from 10 mg/L of CuO NPs in the presence or absence of CNTs; **B**, The effects of released Cu^2+^ on denitrification in marine sediments. Error bars represent standard deviations of triplicate tests.

**Supplementary Figure S2**

**
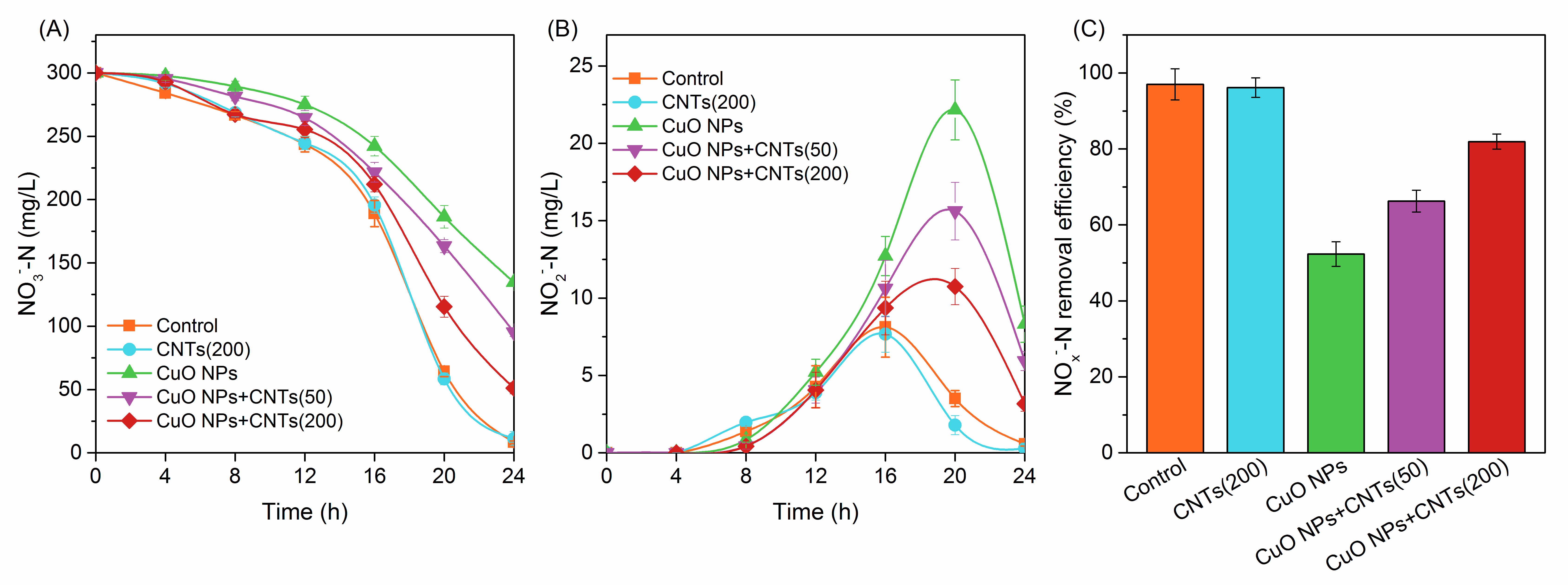
**

**Figure S2 | Effects of CNTs on the toxicity of CuO NPs to denitrifer.** **A**, **B**, and **C** show the variations of NO_3_^-^-N, the accumulation of NO_2_^-^-N, and the final removal efficiency of total NO_X_^-^-N, respectively. Error bars represent standard deviations of triplicate tests.

**Supplementary Figure S3**

**
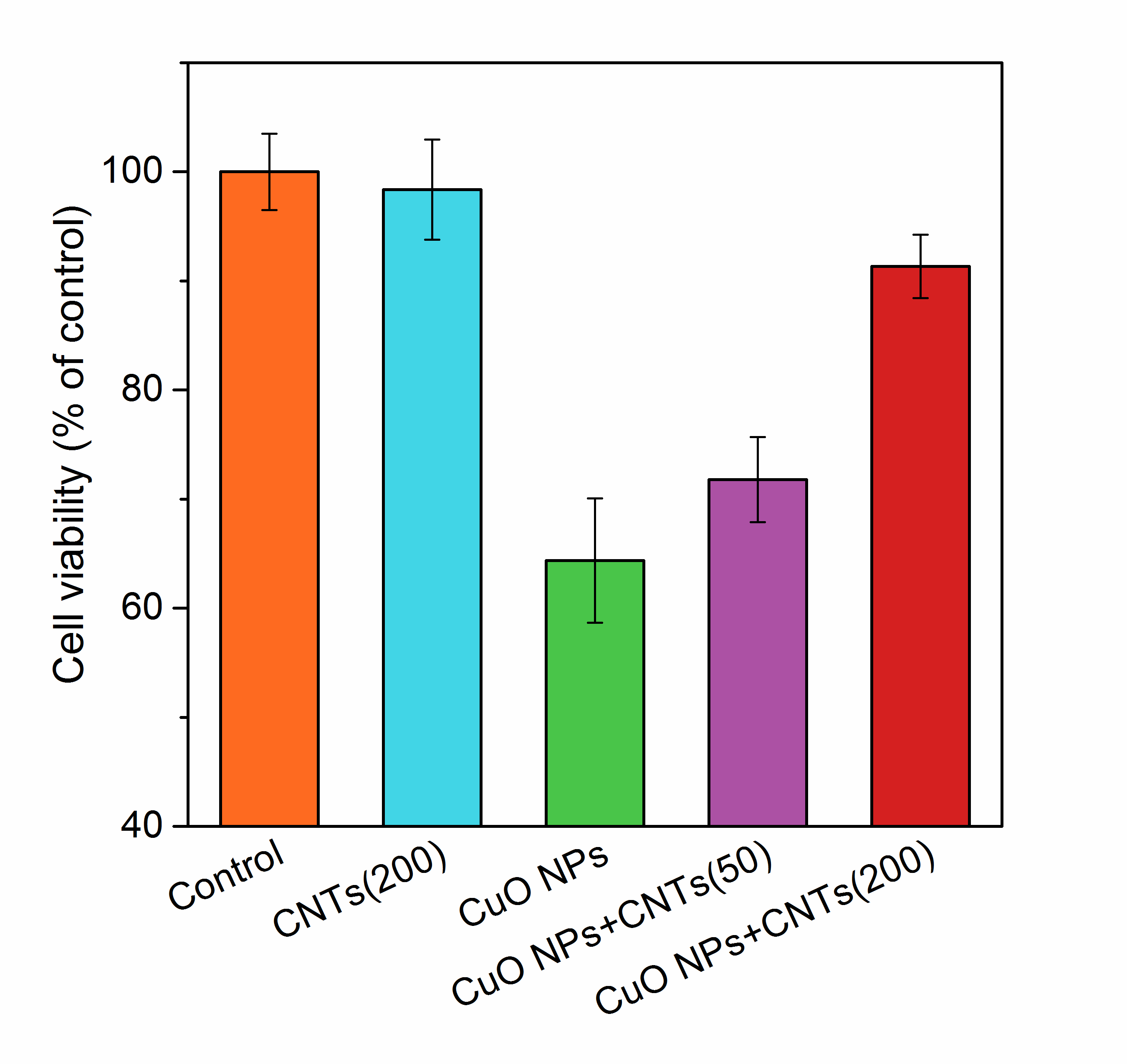
**

**Figure S3 | Effects of CNTs on the toxicity of CuO NPs to cell viability of denitrifier.** Error bars represent standard deviations of triplicate tests.

**Supplementary methods**

**Dissolution of nanoparticles and its influence on denitrification.** The ion release of CuO NPs in the medium was determined according to previous publications^50, 51^, and the purified CNTs did not release free ion after the removal of metal catalyst residual^52^. The serum bottles contained 10 mg/L CuO NPs in 50 mL of the mineral medium with the presence of 0, 50 and 200 mg/L CNTs, and then shaken at 30 ^o^C and 200 rpm. After equilibration for 1, 4, 12 and 24 h, the supernatant was gotten by high speed centrifugation (12000 rpm) for 20 min, and digested with 2% nitric acid (HNO_3_) to remove organic matter. The concentration of Cu^2+^ in the resulting solution was determined using an Agilent 720 ICP-ES (Agilent, USA). Then, the sedimentary inocula in mineral medium was exposed to the corresponding concentrations of Cu^2+^ according to the exposure experiment of nanomaterial, and the concentrations of NO_3_^-^-N and NO_2_^-^-N were measured.

**Supplementary references**

50. Atha, D.H. et al. Copper oxide nanoparticle mediated DNA damage in terrestrial plant models. *Environ. Sci. Technol.* **46**, 1819-1827 (2012).

51. Wang, Z. et al. CuO nanoparticle interaction with human epithelial cells: Cellular uptake, location, export, and genotoxicity. *Chem. Res. Toxicol.* **25**, 1512-1521 (2012).

52. Liu, Y., Zhao, Y., Sun, B. & Chen, C. Understanding the toxicity of carbon nanotubes. *Accounts Chem. Res.* **46**, 702-713 (2013).
